# Single-Cell Sequencing Technology in Oncology: Applications for Clinical Therapies and Research

**DOI:** 10.1155/2016/9369240

**Published:** 2016-05-25

**Authors:** Baixin Ye, Qingping Gao, Zhi Zeng, Creed M. Stary, Zhihong Jian, Xiaoxing Xiong, Lijuan Gu

**Affiliations:** ^1^Department of Hematology, Renmin Hospital of Wuhan University, Wuhan, Hubei 430060, China; ^2^Department of Pathology, Renmin Hospital of Wuhan University, Wuhan, Hubei 430060, China; ^3^Department of Anesthesiology, Perioperative and Pain Medicine, Stanford University School of Medicine, Stanford, CA 94305, USA; ^4^Department of Neurosurgery, Renmin Hospital of Wuhan University, Wuhan, Hubei 430060, China; ^5^Central Laboratory, Renmin Hospital of Wuhan University, Wuhan, Hubei 430060, China

## Abstract

Cellular heterogeneity is a fundamental characteristic of many cancers. A lack of cellular homogeneity contributes to difficulty in designing targeted oncological therapies. Therefore, the development of novel methods to determine and characterize oncologic cellular heterogeneity is a critical next step in the development of novel cancer therapies. Single-cell sequencing (SCS) technology has been recently employed for analyzing the genetic polymorphisms of individual cells at the genome-wide level. SCS requires (1) precise isolation of the single cell of interest; (2) isolation and amplification of genetic material; and (3) descriptive analysis of genomic, transcriptomic, and epigenomic data. In addition to targeted analysis of single cells isolated from tumor biopsies, SCS technology may be applied to circulating tumor cells, which may aid in predicting tumor progression and metastasis. In this paper, we provide an overview of SCS technology and review the current literature on the potential application of SCS to clinical oncology and research.

## 1. Introduction

Cellular heterogeneity is the characteristic of many cancers [1, 2]. This may be a fundamental result of aberrant stem cell cellular proliferation. The cancer stem cell theory of tumorigenesis describes stem cells as having the potential to develop into different subgroups of cancer cells with unexpected phenotypic characteristics [3]. During tumorigenesis, harmful gene mutations may be selected via adaptation to the varied tumor microenvironment. Therefore the genomic profile of many cancers can be considered dynamic. This likely contributes to immune evasion and resistance to chemotherapy [4]. The most effective current cancer therapies appear to be correlated with high degrees of cellular homogeneity within the tumor [5]. For example, acute promyelocytic leukemia (APL, the M3 subtype of acute myeloid leukemia) can be largely cured by the drugs all-*trans*-retinoic acid (ATRA) and arsenic trioxide (ATO) in combination. This effect is most likely achieved by effectively targeting the oncogene PML-RAR*α* protein homogeneously expressed in nearly all APL cells. However, for most other cancers, protein expression appears to be significantly heterogeneous, limiting the efficacy of novel targeted therapies [1].

The individual cell is the fundamental unit of all physiologic tissue. Thus, understanding the cellular evolution and genomic variability of cancers and tumor subtypes at the single-cell level is a critical step in the development of “personalized” cancer therapies [6, 7]. The rapid advancement of single-cell sequencing (SCS) technology has become an invaluable tool to define and characterize the genomic, transcriptomic, and epigenomic heterogeneity in cancer development [8]. For example, by employing SCS to circulating tumor cells, metastasis and progression diagnoses may aid in therapeutic design and enhanced eradication of tumors with different cellular subpopulations [9, 10]. In this review, we will introduce the general procedures of SCS and describe how the generation of genomic, transcriptomic, and epigenomic profiles will provide a framework for the technological advancement of oncological research and ultimately promote the development of novel therapies for cancer.

## 2. Procedures and Methods of Single-Cell Sequencing (SCS) Technology

**Figure 1:**
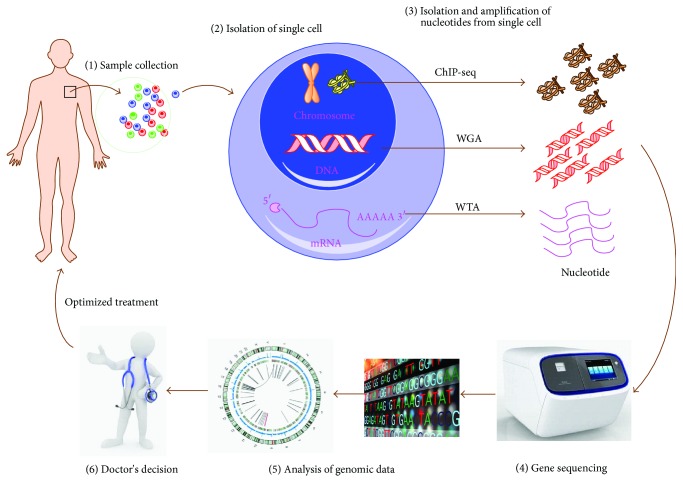
Procedures of single-cell sequencing (SCS) technology in cancer treatment. (1, 2) The patient's sample is collected and then the single cell is isolated from the sample by means of serial dilution, mouth pipetting, flow sorting, robotic micromanipulation, and/or microfluidic platforms. (3) The nucleotide of the single cell is isolated and amplified by specific methods such as whole-genome amplification (WGA), whole-transcriptome amplification (WTA), and ChIP precipitation, which permit analysis at the genomic, transcriptomic, and epigenomic level. (4, 5) The amplified nucleotide is sequenced by a gene sequencer and the information is analyzed using bioinformatic methods. (6) The “omics” data can aid the clinician to determine an optimized treatment strategy.

The development of the first Next-Generation Sequencing (NGS) technology in 2005 provided the novel possibility of performing genome-wide single-cell sequencing [8]. Single-cell RNA sequencing was first described in 2009 [11], and following that, the first single-cell DNA sequencing was described in 2011 [6]. These groundbreaking developments were followed by the first descriptions of epigenomic sequencing in 2013 [12]. The procedures of single-cell sequencing can be simplified to include sample collection, single-cell isolation, nucleotide sequence (DNA or RNA) amplification, and DNA sequencing and data analysis (Figure 1). In the following we will discuss the general procedures associated with single-cell sequencing technology.

### 2.1. Sample Collection and Single-Cell Isolation for SCS

The initial step for SCS is isolation of the single cell of interest from the sample. Single-cell samples have traditionally been obtained from biopsies of the tumor tissue or body fluids, including blood, brain fluid, and urine [8]. To isolate the single cell from an abundant population of cells randomly, the following methods have been described: serial dilution, robotic micromanipulation, flow-assisted cell sorting (FACS), and microfluidic platforms [8, 13]. These methods require that the cells of interest be isolated from fresh tissues and then prepared in suspension. Therefore, samples which have been flash-frozen or formalin-fixed and paraffin-embedded can not be used for single-cell isolation. The limitation of these methods includes technical mastery, a high probability of isolating multiple cells, and low throughput. As such, when the number of cells of interest is rare (<1%), isolation of single cells can be exceedingly difficult. Standard methods of cell isolation have been modified to improve resolution [14], and many novel strategies to address this difficulty have been developed, such as Nanofilters*™*, MegSweeper*™*, CellSearch*™*, CellCelector*™*, and DEPArray*™*. In detail, Nanofilters technology has been employed to discriminate and select individual cells of interest based on a specific cellular size [15]. MegSweeper and CellSearch employ magnetic beads with specific antibodies to “fish” cells of interest that express special membrane markers [16, 17]. The DEPArray system applies a charge to select single cells of interest via a microchip with dielectrophoretic cages. At a cost, the aforementioned methods have the limitation that the spatial information of a single cell is missed. To overcome this, laser-capture-microdissection (LCM) has been used to preserve the context of single cells in the spatial dimension by dissecting the single cell of interest in tissue section [18]. However, the operation of cell slicing and UV damage in RNA/DNA make LCM have the possibility of influencing the sequencing result. Therefore, none of the currently developed methods are without limitations. Individually selecting and utilizing combinations of methods can be employed to minimize the specific limitations of each technique. Another limitation is the reproducibility of sequencing. For example, the genome of a single cell even in the same cell line can be different, and therefore the sequencing result of single cells may not be replicated completely. Additionally, the quantity of nucleotide derived from a single-cell sample creates technical difficulties in producing reliable replicates. Therefore, future techniques will need to be developed to overcome these limitations.

### 2.2. Methods for Amplification of Single-Cell DNA and RNA

Individual cells contain ~6 pg DNA and ~10 pg RNA, and these limited quantities stretch the capabilities of DNA or RNA sequencing [8]. Thus, in order to obtain more nucleotides for DNA library construction, genome-wide nucleotide amplification including whole-genome amplification (WGA) and whole-transcriptome amplification (WTA) is necessary. For SCS, WGA and WTA provide the basis for the analysis of gene mutations and copy number alterations and the determination of specific cellular gene expression in a single cell. With these techniques, the quantity and quality of amplification of single-cell DNA and RNA are closely related with the result of sequencing. For example, the limited amount of input templates for WGA or WTA leads to a number of technical errors, including false positive and false negative errors, coverage nonuniformity, and allelic dropout (ADO) events. Therefore, using proper methods to promote and validate the WGA and WTA output in an unbiased manner is critical in successful nucleotide sequencing.

For DNA SCS, WGA provides sufficient nucleotide for sequencing library construction [6, 19]. There are currently three methods that have been described for WGA: degenerative-oligonucleotide-PCR (DOP-PCR), multiple-displacement-amplification (MDA), and multiple annealing- and looping-based amplification cycles (MLBAC) [7, 12, 20–22]. DOP-PCR is the first method developed for SCS, which employs hybrid oligonucleotides containing both degenerate and defined sequences to amplify DNA, in both semirandom and nonrandom priming manners. DOP-PCR can produce low physical coverage (10%) of a single-cell genome. This allows the process to work well in high-resolution copy number profiles but is limited by low-resolution when measuring mutations at the nucleotide base level [6, 20]. The second common method for WGA is MDA, which helps synthesize DNA fragments via the denatured single-cell DNA template, followed by displacement of the former DNA fragment with the latter newly synthesized fragment. This process liberates the single-stranded DNA for new primer annealing and DNA-synthesis. MDA has been widely reported to achieve high physical coverage (>90%) from a single-cell genome or exome, which is ideal for measuring base mutation but poor for measuring DNA copy number [7, 21]. The third WAG method, MLBAC, polymerizes circular DNA fragments followed by adaptor ligation of PCR. This produces both DNA copy number data and information about single-nucleotide variation [22]. In comparison, for genomic DNA sequencing the library preparation does not require prior WGA. Therefore, genomic DNA isolation from the cell population of interest is followed by library construction. Given that the process of WGA methods discussed here can lead to bias caused by DNA amplification, SCS should be performed by selecting the appropriate method(s) according to the practical needs.

Whole-transcriptome (messenger RNA, mRNA) amplification is a critical step for successful appropriate SCS [19, 23]. The initial step for RNA sequencing is to develop effective methods for whole-transcriptome amplification [8, 19, 23]. In the past five years, RNA sequencing has gained much advancement. At the initial stage of WTA, polyadenylated mRNA is reversely transcribed and selectively amplified using oligo-dT primers conjugated to an adaptor sequence. This produces complementary DNA (cDNA) by oligo-dT anchoring and template switching methods. Next, PCR or in vitro transcription (IVT) methods are used to amplify cDNA for the following sequencing. Until now, classic WTA methods have been modified: this incudes single-cell transcriptional landscape by highly multiplex RNA-seq (STRT [24, 25], “Smart-Seq” (Smart-Seq2 [26]), and single-cell RNA-seq by multiplexed linear amplification CEL-seq/MARS-seq [27]). As an unavoidable consequence, these methods employ a strong 3′ mRNA amplification bias.

### 2.3. Epigenetic Considerations

The concept of* epigenetics* describes functionally relevant changes to the genome that do not involve a change in the nucleotide sequence of nontumorigenic single cells [28]. Methods borrowed from epigenetic profiling may more accurately describe the genomic fate of the individually isolated tumor cell [29]. The epigenome contains the landscape of all epigenetic marks that exist in a cell and their chromosomal manifestations [30]. This effect defines every stage of cellular development and cancer progression [31]. To uncover the secrets defining each individual single cell at the genomic level, epigenomic sequencing is critical. However, methodologically, it is very challenging to precisely describe and define the epigenomic markers in the DNA sequence or chromatin composition [8]. The most common standard epigenomic sequencing methods include (1) bisulfite sequencing (BS-seq) and (2) chromatin immune-precipitation followed by sequencing (ChIP-seq) for analyzing histone modification and chromatin binding proteins [31]. For BS-seq, the pool of DNA requires separation into two parts for treatment with bisulfide or methylation restriction enzymes prior to sequencing. In addition, the epigenetic modifications of DNA cannot be amplified by polymerases. In essence, it is a formidable technical challenge to accurately characterize the single-cell epigenomic landscape [8]. Recently, it is reported that reduced single-cell representation bisulfite sequencing (scRRBS) could measure cytosine methylation modification in a single cell at about 10% of the genome [32]. In another study, the single-cell ChIP-seq method was developed by combining microfluidics, DNA barcoding, and sequencing to collect chromatin data at single-cell resolution, depicting high-quality chromatin state maps and thus defining the subpopulation of cell types [29]. Most promisingly, a method of parallel single-cell genome-wide transcriptome and methylome sequencing was recently developed, which may reveal the linkage between heterogeneously methylated distal regulatory elements and transcriptional activity of major pluripotency genes in 61 mouse embryonic stem cells at a single-cell level [33]. Therefore, it appears that methods with higher resolution and additional dimensions for single-cell sequencing may provide a future path towards epigenomic sequencing of individual cancer cells and more personalized oncological treatments.

### 2.4. Gene Sequencing and Data Analysis

Developed in 2005, the NGS platform rapidly reduced the cost and time needed for human genomic sequencing. This provided a powerful tool to serve in the diagnosis and treatment of a plethora of diseases [34]. However, given multiple inherent technical errors that exist in current single-cell sequencing technologies, improved bioinformatic analysis is a critical requirement for differentiating noise from meaning. With the continued advancement of NGS technology and bioinformatics, promising developments may be applied to SCS technology and their applications to personalized oncological therapies.

## 3. Application of SCS in Cancer Treatment and Research

### 3.1. SCS May Reveal Evolutionary Structure and Heterogeneity of Tumor Cells

**Figure 2:**
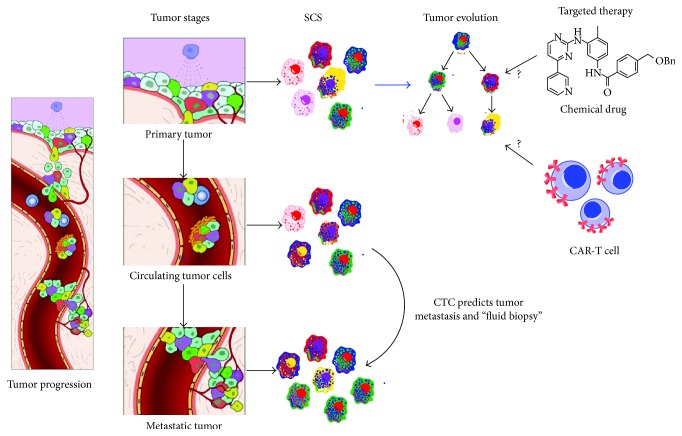
Application of single-cell sequencing technology in tumor evolution and metastasis. In tumor progression, some tumor cells at the primary site can invade into the basement of blood vessels, migrate into the blood circulation, and then implant in the distant tissue site, defined as metastasis. Circulating tumor cells (CTCs) can be easily collected and single-cell sequencing of CTC can be used to predict tumor metastasis. Determining the driver gene mutations of cellular subpopulations may aid in understanding on the tumor evolution and the development of targeted therapy using chemical drugs and immune cell therapies, such as chimeric antigen receptor modified-T cells (CAR-T cells).

Cancer development is characterized by fundamental changes in gene expression. This change may be considered* cancer evolution,* as it involves the process of mutational diversification and clonal selection [10]. Characterization of the mechanisms that determine such genomic changes may provide a quantum leap in cancer diagnosis and prognosis and the development of novel effective therapies [35, 36]. In the temporal and spatial dimensions of cancer development, multiple genomic changes may occur in the same tumor in adaptation to local environmental cues. This characteristic likely contributes to the heterogeneity of cancer in both phenotype and genotype and may hold keys to determining the mechanisms of alterations in cell fate and metastasis. The development of SCS technology provides a means to dissect the evolutionary structure and identify the activating genes in cancer development at the single-cell level (Figure 2). For example, in 2011 the technology of single nucleus sequencing was used to study tumor cell population, structure, and evolution in two breast cancer patients by analyzing genome copy number variation [6]. In this study, the nuclei of 100 single cells isolated from the primary tumor sites and metastatic sites were extracted and single-cell DNA was amplified and sequenced. Utilizing bioinformatic analysis on the copy number variation, the results indicated that a single clone expanded into the primary tumor and further seeded to the metastasis.

Subsequently in 2012, the MDA method of WTA was developed, permitting analysis of the cancer genome at a single-cell nucleotide level [7, 21]. Hou et al. sequenced and analyzed the genome of 90 single cells from a JAK2-negative myeloproliferative neoplasm essential thrombocythemia (ET) patient. Their results suggested that ET represented a monoclonal evolution and identified the driver genes SESN2 and NTRK1. Xu et al. analyzed cells from clear cell renal cell carcinoma, a common kidney cancer that shares few mutations among different patients. They described a detailed intratumoral genetic landscape at a single-cell level [21]. These results indicate that SCS methodology can be applied to individual cancers and provide a framework for the development of more effective cellular targeted therapies. Versus traditional cellular assays, SCS has the advantage to identify rare driver genes existing in the clone of tumors, which could not otherwise be observed in the general cellular population. As an example, a recent study by Yu et al. [37] analyzing colon cancer cells demonstrated that the driver oncogene SLC12A5 mutation could be identified at the single-cell level but could not at the cell-population level. Additionally, single-cell RNA sequencing (RNA-seq) was also applied to define the heterogeneity of cancer cells. Patel et al. used single-cell RNA-seq method to profile 430 cells from five primary glioblastomas and define its intratumoral heterogeneity with potential prognostic implications [38]. This suggested that uncovering the previously unappreciated heterogeneity may help characterize cancer prognosis and therapy [38].

### 3.2. SCS of Circulating Tumor Cells Can Predict Tumor Metastasis and Progression

Circulating tumor cells (CTCs) compose a rare tumor type (1 in 1 million) that originate from primary tumors and migrate to other sites via the circulation [8, 9, 14]. CTCs contribute to metastasis, thereby leading to the majority of mortality in cancer patients. Individual genomic analysis and characterization of primary tumor cells, CTCs, and metastatic cells may provide mechanistic insight into activation of CTCs [39]. In order to uncover the genomic linkage between primary tumor, CTCs, and metastatic cells, single CTCs can be isolated and subjected to WGA, followed by genome-wide DNA sequencing. Given that CTCs in different types of cancers are varied, CTCs define a cellular population that is inherently heterogeneous and rare in the circulation. Therefore, isolating populations of CTCs with a repeatable, reliable, rapid, cost-effective, and automated method is a significant challenge [40–42]. Presently, three methods have been developed for CTC isolation: immunoaffinity, isolation based on physical properties, and direct analysis. Immunoaffinity-based methods refer to the use of magnetic beads or nanostructured substrates (silicon nano/micropillars) with specific antibodies to target specific markers, thus selectively enriching CTCs or depleting leukocytes. This method is widely used in CTC isolation: CellSearch for CTCs quantification has been approved clinically in metastatic breast, prostate, and colon cancers. The primary limitation of this approach is antigen-antibody specificity and long interaction times. An alternative approach, selection based on physical properties, selects CTCs by density, size, deformability, and electrical properties. These methods utilize density gradient centrifugation, microfiltration, microfluidics, and dielectrophoresis, which are all independent of cell antigen expression but limited by CTC purity. The third approach, direct analysis, utilizes fiber-optic scanning and Hall effect sensing, achieved by high throughput assaying of all cells in the blood after erythrocyte lysis. This method holds promise for CTC detection because of less vulnerability to cell loss with sampling but is limited by poor CTC recovery. Thus, the current methods available to isolate CTCs from cancer patients will benefit from further technological advances to eliminate the individual limitations.

As CTCs can be isolated from the patient's blood, genome-wide sequencing of CTCs can offer a noninvasive measure to optimize the diagnosis and even prognosis of cancers [14]. At the genome-wide transcriptomic level, the single-cell RNA sequencing method (Smart-Seq) has been applied to dissect the distinct gene expression patterns in the single melanoma CTC, promoting the identification of gene biomarkers of CTCs [26, 43]. Using targeted DNA sequencing methodology, Heitzer et al. demonstrated in colon cancer that many driver mutations in the primary tumor are also found in the CTCs, suggesting that the genomic analysis of CTCs may represent the mutational pattern of the evolution of a tumor [44]. This study provides support for the application of SCS to CTCs isolated from the blood of patients, effectively constituting a tumor “liquid biopsy.” Ni et al. demonstrated in single CTCs from lung cancer patients single-nucleotide variations and insertions/deletions in exomes, the region of the genome that remains within the mature RNA after introns are removed by RNA splicing [39]. They found that the copy number variation of CTCs is cancer-specific and is closely related to tumor metastasis. Lohr et al. similarly observed in prostate cancer the coincident recurrence of mutations in primary tumor, CTCs, and metastatic tissue [45]. Thus, single-cell sequencing of CTCs provides a window to observe the intratumoral or metastatic tissues at the genomic or transcriptomic level and may aid oncologists to noninvasively track cancer mutational evolution and provide optimized therapeutic strategies prior to the emergence of drug resistance [9].

### 3.3. The Potential Role of SCS Technology in Personalized Medicine

Precision medicine defines the future of personalized medicine [46]. Because several oncogenic mutations can occur in the same patient, serial genomic analysis of the individual patient over time will be an important component [47]. An important example of this was demonstrated in a recent study by Engle et al. [48]. They analyzed cells in a patient with primary myelofibrosis (PMF) transformed to secondary acute myeloid leukemia (sAML). The samples of the patient at the diagnosis of PMF and sAML were collected, followed by WGS. The architecture of clones during the transformation of MPN to sAML was characterized, suggesting potential novel treatments for patients who demonstrate this phenotype. In the temporal dimension, the analysis of clone architecture of cancer cell evolution at the single-cell level would promote a deeper understanding of the order of genetic events that occur within the cancer genome. In a recent study, Gawad et al. analyzed 1,479 single tumor cells from six acute lymphoblastic leukemia (ALL) patients with SCS, observing sequential deletions, single-nucleotide variants (SNVs), and IgH sequence changes [49]. Their observations at a single-cell resolution suggested that the sequence of genetic events that underlie childhood ALL occurs in order. Thus, from the clonal evolution perspective, single-cell sequencing technology will likely be a powerful weapon to help clinicians better understand the pathogenesis of diseases at a high-resolution and provide individualized treatments specifically optimized for their patients [4, 10].

Somatic evolution during tumor development is closely associated with the accumulation of genetic (and epigenetic) mutations. By identifying the contributions to tumor cell proliferation and invasion, somatic mutations can be characterized as “driver” or “passenger” in the context of Darwinian dynamics [50]. Driver mutations can lead to significant tumor response. Therefore, identification of driver mutations underlying tumor progression may aid in the development of personalized medicine. However, because driver gene mutations are usually transient and change upon the emergence of the new resistant phenotype, targeting more common “druggable” driver mutations may be a necessary approach. However this approach would decrease the selective phenotypic characterization, decreasing the degree of personalized therapy. Recently, it has been proposed from observations made from gene sequencing of cancers that the mode of tumor evolution occurs in a non-Darwinian manner [51]. Driver gene mutations can be considered to compose a phylogenetic “tree,” which contains “trunk” and “branch” mutations in both temporal and spatial dimensions [10, 52]. In this phylogenetic tree, trunk mutations are defined as gene mutations that all cells gain. In comparison, branch mutations are gene mutations gained by some subgroups of cancer cells [53]. In theory, the phenotypes of evolved cancer cells such as metastasis, drug resistance, and relapse are attributed to genetic mutations at either or both of these levels. It has been proposed that trunk genes and branch mutations may be differentially controlled [54]. Therefore, defining the genomic characteristics of the phylogenetic tree is a critical step in identifying the potential driver genes to target with drugs or immune cells for effective therapy. Recent advancements with immune therapy utilizing chimeric antigen receptor T cells to target cancer cells show very promising results [55]. This powerful technology can potentially be employed to target specific tumor antigens identified by SCS.

## 4. Conclusions

The great success of chronic myeloid leukemia treatment by imatinib and promyelocytic acute leukemia treatment by ATRA and ATO showed that targeting the important oncogenetic mutation is promising for curing cancers [5, 56]. However, heterogeneity of genotype and phenotype is a hallmark characteristic of the temporal and spatial progression of tumor development, making targeted oncological therapies difficult. SCS methods provide a mechanism to investigate the evolutionary structure of tumors and the genomic information of rare cell populations such as CTCs. Recent advancements in SCS technology permit new characterizations of tumor transformation, metastasis, chemoresistance, antigenicity, and immunoediting. SCS technology therefore provides a more detailed understanding of the genomic architecture of cancer cell subpopulations, promoting the development of novel tumor-targeting strategies in the context of intratumor heterogeneity. However, the high price of gene sequencing, difficulty in isolation and identification of single cells of interest, and the computational analysis of sequencing data remain obstacles to the application of SCS technology to the oncologic clinic [8]. Developing a complete understanding of the clinical significance of trunk and branch mutations in the cancer phylogenic tree and subsequent development of targeted therapies will require extensive future investigation in both the laboratory and clinic.
